# Recurrent Corneal Erosion Syndrome in Epithelial Basement Membrane Dystrophy: Current Treatments and Mechanism-Guided Therapeutic Opportunities

**DOI:** 10.3390/cells15201912

**Published:** 2026-10-21

**Authors:** Sujin Kang, Alfonso L. Sabater, Sotiria Palioura, Sonia H. Yoo

**Affiliations:** Bascom Palmer Eye Institute, University of Miami Miller School of Medicine, Miami, FL 33136, USA; sxk2340@miami.edu (S.K.); asabater@med.miami.edu (A.L.S.); spalioura@miami.edu (S.P.)

**Keywords:** recurrent corneal erosion, epithelial basement membrane dystrophy, anterior stromal puncture, diamond burr polishing, phototherapeutic keratectomy, regenerative therapy

## Abstract

Recurrent corneal erosion syndrome (RCES) and epithelial basement membrane dystrophy (EBMD), also termed anterior basement membrane dystrophy, are distinct entities. RCES most often follows shearing corneal trauma, whereas EBMD may be asymptomatic, may blur vision without pain, or may predispose to erosions through defective epithelial attachment. This narrative review focuses on RCES associated with EBMD. It emphasizes how treatment choice depends on clinical findings, on whether the main goal is pain control, recurrence prevention, or visual rehabilitation, and on how directly the evidence applies to EBMD rather than to mixed-cause erosions. Lubrication and bandage lenses protect the healing surface, while anti-inflammatory and blood-derived therapies support repair. Debridement removes unstable epithelium; manual superficial keratectomy, diamond burr polishing, alcohol delamination, and phototherapeutic keratectomy treat the abnormal interface. Anterior stromal puncture provides focal scar-mediated anchoring, principally outside the visual axis. Contemporary procedural studies support individualized treatment but do not establish a universally superior technique. A heparan sulfate mimetic polymer has been tested directly in EBMD, but only in one small uncontrolled series; topical insulin, neurotrophic treatments, and differentiation-related targets lack EBMD-specific evidence of benefit. Epithelial closure, prevention of painful erosions, and optical rehabilitation are distinct outcomes and should not be treated as interchangeable measures of success. Management of EBMD-associated RCES should therefore be guided by the dominant clinical problem, lesion location, and the specific therapeutic outcome sought.

## 1. Introduction

Recurrent corneal erosion syndrome (RCES) is characterized by repeated episodes of corneal epithelial breakdown, typically causing episodic pain, tearing, photophobia, and blurred vision, often on awakening [1]. Although RCES most commonly follows superficial shearing trauma to the cornea, such as a fingernail or paper-edge injury, epithelial basement membrane dystrophy (EBMD), also known as anterior basement membrane dystrophy (ABMD) [2], is another important predisposing condition [1,3]. RCES and EBMD are distinct but overlapping clinical entities. Many patients with EBMD remain asymptomatic [4]. When symptoms occur, patients may present with painful recurrent erosions, blurred or fluctuating vision caused by an irregular epithelial surface, or both [4,5,6]. These presentations have different therapeutic goals: treatment of erosions aims to prevent epithelial breakdown and pain, whereas treatment of visually significant EBMD aims to improve corneal regularity and optical quality.

This review focuses specifically on RCES arising in the setting of EBMD. The available evidence is difficult to interpret because RCES studies frequently combine traumatic and dystrophic cases, while EBMD studies often combine eyes treated for erosions with those treated for visual symptoms. We therefore evaluate the literature according to both the etiology of RCES and the indication for treatment, separating EBMD-associated erosion outcomes whenever possible. Because many of the same procedures are used to treat visually significant EBMD, their optical outcomes are discussed separately and are not considered evidence of erosion control. Finally, we examine how current insights into epithelial adhesion and wound healing may inform future treatment strategies for EBMD-associated RCES [7].

## 2. Clinical Assessment and Treatment Goals

Evaluation begins with a careful history and bilateral slit-lamp examination. The history should document the frequency and timing of episodes, morning pain, sleep interruption, visual symptoms, previous treatments or procedures, and associated dry eye or meibomian gland dysfunction. Clinical examination with direct illumination, retroillumination, and fluorescein staining may reveal map-like changes, fingerprint lines, microcysts, loose epithelium, negative staining or an active epithelial defect (Figure 1) [4]. The location and extent of disease should be recorded as central or peripheral and focal or diffuse because these features may influence procedural selection. A history of corneal trauma does not exclude EBMD, since trauma may precipitate erosions in an eye with an underlying epithelial adhesion abnormality [2,3].

RCES has multiple etiologies, making it essential to identify features implicating EBMD before selecting erosion-directed treatment. In a series of 100 patients with RCES, previous superficial trauma and EBMD were the two most common identifiable causes [8]. EBMD-associated erosions are suggested by spontaneous onset or onset after trivial contact, recurrence at different corneal sites or in both eyes, and map, dot, or fingerprint changes in either eye. In contrast, post-traumatic erosions typically follow a discrete shearing injury, recur at the original injury site, and exhibit a normal surrounding cornea between episodes [1,3,8,9]. Negative fluorescein staining over loose epithelium and subepithelial hyperreflective material or irregular epithelial thickness on AS-OCT further support EBMD, whereas post-traumatic erosions typically show localized irregularity without diffuse abnormalities [10,11,12].

Histopathologically, EBMD is characterized by a thickened, multilaminar basement membrane extending into the epithelium, intraepithelial microcysts, and basal epithelial cells resting on an aberrant basement membrane. In contrast, post-traumatic erosions exhibit basement membrane attenuation confined to the site of injury [2,5,13,14]. Ultrastructural examination of alcohol-delaminated epithelial sheets further distinguishes these mechanisms: trauma-related erosions separate within the lamina lucida, whereas EBMD-associated erosions involve removal of the entire basal lamina with the epithelial sheet (see Section 5.4) [15]. These etiologic categories are not mutually exclusive, as minor trauma may unmask underlying EBMD. Both the history of trauma and clinical signs of EBMD should therefore be documented [2,3].

Alternative or coexisting causes of epithelial breakdown should be considered, including herpetic or microbial keratitis, retained foreign body, exposure keratopathy, and neurotrophic keratopathy. Reduced corneal sensation, a stromal infiltrate, atypical staining, or delayed epithelial healing should prompt reassessment of the diagnosis before erosion-directed treatment is escalated [3].

Corneal topography and epithelial thickness mapping are useful adjuncts when blurred or fluctuating vision is prominent or when cataract or refractive surgery is planned. Corneal topography can document irregular astigmatism and corneal surface distortion in visually significant EBMD and identify surface abnormalities that may compromise the reliability of preoperative keratometry and intraocular lens power calculations [16]. Epithelial thickness mapping can demonstrate regional epithelial thickening and increased thickness variability in EBMD. In vivo confocal microscopy (IVCM) can reveal hyperreflective material within the basal and intermediate epithelial layers, epithelial microcysts, and alterations of the subbasal nerve plexus; however, its contact-based, operator-dependent nature limits routine clinical use [10]. A cross-sectional optical coherence tomography study found greater epithelial thickness irregularity in eyes with EBMD than in eyes with dry eye disease or healthy controls but did not assess whether these findings predict erosion recurrence or treatment response [11]. Imaging should therefore complement rather than replace clinical phenotyping.

Early-onset or familial recurrent corneal erosions warrant consideration of distinct inherited disorders, including *COL17A1*-associated epithelial recurrent erosion dystrophy [17]. The third edition of the International Classification of Corneal Dystrophies classifies EBMD as a category 3 disorder (i.e., a well-defined corneal dystrophy that has not been mapped to a chromosomal locus), while acknowledging that most cases appear to be degenerative rather than inherited [2].

Establishing shared treatment goals before escalation can help clinicians and patients decide whether the priority is acute pain relief, prevention of future erosions, or improvement in optical quality. These goals may overlap, but they require different measures of success. In an eye with an active erosion, the immediate priorities are epithelial healing and pain control; after closure, the goal becomes prevention of recurrence. In patients whose primary complaint is blurred or fluctuating vision without painful erosions, treatment instead aims to improve epithelial regularity and optical quality (see Section 6).

## 3. Adhesion Biology Relevant to Clinical Care

Stable epithelial adhesion depends on the epithelial basement membrane (EBM) and its attachment to Bowman layer and anterior stroma [13,14,18,19,20,21]. Basal epithelial hemidesmosomes, including α6β4 integrin and collagen XVII, bind laminin-332; collagen VII anchoring fibrils connect the EBM to underlying tissue (Figure 2). The normal EBM also contains a collagen IV network, including α1α1α2 and α3α4α5 isoforms, linked to laminins by nidogens and the heparan sulfate proteoglycan perlecan [13,18]. In EBMD, excessive, multilayered basement membrane material extends into the epithelium: broad subepithelial sheets produce geographic maps, redundant folds produce parallel fingerprint lines, and entrapped degenerating epithelial cells form intraepithelial microcysts seen as dots [2,4,5]. Displaced basement membrane separates basal cells from their usual substrate and disrupts hemidesmosomal anchoring, leaving epithelium vulnerable to lid-induced shear and recurrent erosion [2,13].

### 3.1. Molecular Basis of Defective Epithelial Adhesion in EBMD

The molecular basis of defective epithelial adhesion in EBMD remains incompletely understood. Unlike inherited epithelial adhesion disorders caused by defects in specific structural proteins, such as *COL17A1*-associated epithelial recurrent erosion dystrophy, EBMD appears to involve dysregulated basement membrane deposition and organization rather than loss of a single adhesion component [2,13,17]. Although most cases are considered degenerative, heterozygous *TGFBI* mutations have been identified in a subset, suggesting genetic heterogeneity [2,22]. Reduced expression of PAX6 and desmoglein-1 in affected epithelium further implicates altered epithelial differentiation and cell–cell cohesion, although these associations do not establish causality [23]. The resulting adhesion defect is therefore likely to reflect abnormalities in both basement membrane architecture and epithelial cell function. This distinction provides a mechanistic rationale for treatments that modify the abnormal epithelial basement membrane interface and promote stable reattachment rather than epithelial closure alone.

### 3.2. Basement Membrane Regeneration After Different Interventions

Epithelial closure precedes restoration of durable attachment. When the EBM remains intact after a scrape wound, regenerating epithelium can reuse the preexisting basement membrane and re-establish firm adhesion within several days. In contrast, after keratectomy-type injury that removes the EBM, reassembly of the basement membrane and associated adhesion structures, including hemidesmosomes and anchoring fibrils, proceeds over weeks and may remain incomplete for months [14,19]. Experimental wound-healing studies further show that epithelial cells deposit a nascent laminin scaffold, while keratocytes and fibroblasts contribute nidogen-1, nidogen-2, and perlecan to EBM assembly [18,24]. These differences help explain why epithelial closure does not immediately restore durable adhesion and provide a biologic rationale for prolonged surface protection after debridement. However, the timing of complete EBM regeneration varies with injury severity and model, and these intervals should not be interpreted as an EBMD-specific clinical healing timetable [18,24]. The substrate on which the EBM regenerates after each procedure is discussed in Section 5.

The EBM is also a biochemical barrier. Collagen IV, perlecan, and nidogens help restrict stromal access to epithelial- and tear-derived transforming growth factor β (TGF-β) and platelet-derived growth factor (PDGF) [13,18,25]. When the EBM fails to regenerate after epithelial-stromal injury, persistent growth-factor entry promotes development and survival of opacity-producing anterior stromal myofibroblasts; barrier restoration reduces this signaling and permits haze resolution [18,25]. These findings come largely from experimental corneal injury and refractive-ablation models rather than EBMD treatment trials. They provide a mechanistic reason to remove abnormal EBM while limiting unnecessary stromal injury during phototherapeutic keratectomy (PTK), but do not establish a universal safe ablation depth or prove that direct growth-factor modulation prevents EBMD recurrence.

### 3.3. Matrix Metalloproteinases and Interface Remodeling

Matrix metalloproteinases (MMPs) regulate extracellular matrix turnover during corneal epithelial repair but may contribute to defective adhesion when their activity is excessive or prolonged. MMP-2 (gelatinase A) participates in basement membrane remodeling, whereas MMP-3 (stromelysin-1) degrades multiple extracellular matrix components, including laminin, nidogen, perlecan, and collagen IV. MMP-9 (gelatinase B) is induced during epithelial injury and inflammation and may interfere with re-epithelialization when its activity persists [26,27,28].

Evidence from human recurrent corneal erosion studies suggests that MMP involvement varies with disease activity and tissue sampling. Garrana et al. detected MMP-2 activity in 12 of 13 epithelial specimens from patients with recurrent erosions, whereas MMP-9 activity was detected in only one specimen [29]. Increased tear gelatinase activity has also been reported during active erosions and, in some patients, during remission [30]. A subsequent analysis of epithelial specimens collected during remission, including samples from patients with EBMD, demonstrated increased MMP-2 and MMP-3 levels, while MMP-9 was largely undetectable [26,31]. These findings suggest that MMP-9 may be more closely associated with acute epithelial injury, whereas MMP-2 and MMP-3 may contribute to ongoing matrix remodeling. However, their temporal and causal roles in EBMD-associated erosions remain unestablished. Accordingly, MMP-mediated interface remodeling is presented as a proposed mechanism in Figure 2 and Figure 3 rather than a demonstrated disease-specific pathway. The available evidence also does not establish that the clinical benefits of doxycycline or topical corticosteroids in recurrent corneal erosion derive specifically from MMP-9 inhibition.

### 3.4. Implications for Treatment Design

More broadly, the available biologic evidence provides a rationale for several therapeutic strategies: protecting the healing epithelium from recurrent mechanical trauma, controlling inflammation, removing abnormal basement membrane material and renewing the epithelial-stromal interface, creating focal scar-mediated adhesion, and supporting epithelial repair. These mechanisms provide a conceptual framework for understanding existing treatments rather than validated biomarkers or molecular criteria for selecting one intervention over another [18,24,26]. Two therapeutic objectives follow and are not interchangeable: accelerating epithelial closure (lubricants, bandage lenses, blood-derived drops, growth factors, insulin), which acts on migration and proliferation without changing the substrate, and restoring interface architecture (removal of abnormal EBM with regeneration on a preserved or regularized substrate, or scar-mediated anchoring).

## 4. Medical Management

### 4.1. Acute Healing and Recurrence Prevention

Treatment of an acute erosion aims to promote epithelial closure, relieve pain, and reduce the risk of infection. Preservative-free lubrication, topical antibiotic prophylaxis when the epithelium is open, and selected use of cycloplegia or oral analgesia are commonly employed. A bandage contact lens can protect the regenerating epithelium and reduce pain, particularly in patients with a large or persistent defect, but requires antimicrobial prophylaxis and close surveillance for infection [3].

After epithelial closure, daytime lubrication and bedtime ointment may reduce recurrent lid-epithelium friction, particularly during awakening. Hypertonic sodium chloride ointment is another option, although comparative evidence supporting its routine use is limited [7]. In a prospective uncontrolled study of 21 eyes, including 12 with ABMD, treatment with 5% sodium chloride ointment twice daily for 30 days after an acute episode was followed by complete re-epithelialization and symptom resolution in all eyes. One recurrence occurred after new trauma within six months [32]. The absence of a control group makes it impossible to determine whether hypertonic ointment was superior to lubricating ointment or whether the observed improvement reflected the natural healing of the acute episode. Treatment duration and escalation should reflect recurrence burden and tolerability because comparative evidence for conservative regimens remains limited [7].

For patients with continued recurrences despite lubrication, a supervised trial of extended-wear bandage contact lens therapy may precede surgery. In a retrospective series of 12 patients who remained symptomatic despite at least six weeks of lubrication therapy, a bandage contact lens was worn continuously for three months; nine patients (75%) remained free of recurrent erosions during approximately one year of follow-up. Of the three patients who reported recurrent symptoms, only one had objective clinical findings of a recurrent erosion [33]. A 29-patient randomized trial comparing three months of bandage contact lens therapy with ocular lubrication found similar rates of complete resolution at three months (71% vs. 73%, respectively), although symptom resolution occurred earlier with bandage contact lenses (mean, 5 vs. 9 weeks; *p* = 0.02) [34]. These studies support bandage contact lenses as an option for epithelial protection and symptom control, particularly when lubrication alone is insufficient, but have not shown superiority over lubrication for durable prevention of recurrent erosions, nor benefit specific to EBMD-associated disease.

### 4.2. Inflammatory Disease and Impaired Healing

Associated meibomian gland dysfunction and blepharitis should be treated when present because ocular surface inflammation and tear-film instability may contribute to epithelial symptoms and impaired healing. Oral doxycycline with a short course of topical corticosteroid may be considered in selected refractory inflammatory disease after infection has been excluded and with intraocular pressure monitoring [3]. A seven-patient series combined clinical observations with laboratory evidence of MMP-9 inhibition but included no EBMD cases [35]. The reported regimen of doxycycline 50 mg twice daily for two months with corticosteroid treatment for 2 to 3 weeks should therefore not be considered an established EBMD-specific standard.

Autologous serum and platelet-derived eye drops contain epithelial-supporting constituents, including epidermal growth factor, fibronectin, vitamin A, insulin-like growth factor 1 (IGF-1), and TGF-β. The latter is one rationale for diluting serum preparations, although concentrations and clinical effects vary by formulation [36,37]. These therapies support migration and repair but do not remove the abnormal EBM. In a prospective uncontrolled 33-eye series of recurrent erosions unresponsive to previous treatment, autologous serum was used for six months and 28 eyes remained relapse-free during a mean follow-up of 30 months [37]. The study lacked a control group and did not report EBMD-specific outcomes.

A retrospective historical-control study compared 27 eyes treated with platelet-rich plasma plus lubrication with 20 eyes previously treated with lubrication alone [36]. Recurrences were less frequent with platelet-rich plasma, but only three eyes had EBMD, including one treated and two control eyes. Nonconcurrent comparison, cointerventions, and minimal EBMD representation limit applicability. Blood-derived drops may be considered as supportive adjuncts when epithelial recovery is difficult; durable EBMD-specific prevention and incremental benefit after resurfacing remain unproven.

## 5. Procedural Treatment

A procedure may be considered when recurrent erosions remain frequent, severe, or functionally disabling despite an adequate trial of conservative therapy (Table 1, Figure 3). Procedure selection depends on the location and extent of the abnormality, involvement of the visual axis, the presence of optical symptoms, prior treatment, and the principal therapeutic goal. Procedures can be grouped according to their predominant intended effect. Epithelial debridement removes loose or unstable epithelium. Manual superficial keratectomy, diamond-burr polishing, alcohol delamination, and phototherapeutic keratectomy additionally remove or modify the abnormal epithelial basement membrane interface. Anterior stromal puncture (ASP) creates focal scar-mediated adhesion, whereas PTK and other resurfacing procedures may also regularize the central optical surface. These mechanisms overlap, and procedural terminology is not standardized; the operative steps are therefore more informative than the procedure name alone.

The depth of treatment also influences the subsequent repair process (Figure 3). Simple debridement may preserve portions of the existing EBM that serve as a substrate for epithelial reattachment, whereas procedures that remove abnormal basement membrane material through the Bowman layer require deposition and organization of a new adhesion interface [14,19,21]. Restoration of epithelial attachment involves basement membrane assembly and maturation of hemidesmosomes and anchoring fibrils, with contributions from epithelial cells and stromal keratocytes [18,24,47]. IVCM following debridement has documented progressive epithelial reorganization and subbasal nerve recovery, although these changes do not directly establish restoration of durable basement membrane adhesion [48].

PTK may extend tissue removal into Bowman layer and the anterior stroma, producing a repair response that differs from more superficial procedures. Experimental corneal ablation models demonstrate that delayed EBM degeneration can permit sustained stromal exposure to TGF-β and platelet-derived growth factor (PDGF), promoting myofibroblast persistence and haze formation [18,25,49,50,51]. These findings support limiting stromal disruption to that necessary for adequate treatment of the abnormal interface, although an optimal EBMD-specific ablation depth has not been established. Morphologic recurrence after PTK does not necessarily indicate clinical failure [40]. ASP and neodymium:yttrium–aluminum–garnet (Nd:YAG) stromal puncture instead rely on localized fibrosis to reinforce epithelial attachment, making them more applicable to focal peripheral erosions than diffuse abnormalities [52].

Interpretation of procedural studies requires attention to indication as well as etiology. EBMD series commonly include eyes treated for two overlapping indications: painful recurrent erosions and visually significant epithelial irregularity. The same procedure may be used for either indication because both arise from abnormalities of the epithelial basement membrane interface, and both may occur in the same eye [4]. However, reporting by indication is inconsistent.

Aldave and colleagues reported a diamond-burr series in which 37 of 56 eyes were treated for recurrent erosions and 22 for visually significant epithelial irregularity, including three eyes treated for both indications [53]. In the long-term extension by Vo and colleagues, 55 of 91 procedures were performed for recurrent erosions and 42 for visually significant EBMD, with six eyes treated for both [42]. Lee and colleagues similarly reported PTK outcomes separately for 30 eyes treated for visual disturbance and 29 treated for painful erosions [38]. By contrast, some studies report treatment indication but pool subsequent outcomes. Germundsson and colleagues included 18 of 52 eyes treated for erosions, 26 for reduced vision, and 8 for both, but defined clinical recurrence as either recurrent erosion or reduced vision and reported this composite outcome for the entire cohort (7 eyes, 13%) [40]. This distinction is important because erosion-directed studies primarily assess symptoms and recurrence, whereas studies of visually significant epithelial irregularity emphasize visual acuity and corneal surface regularity or topography [38,42]. Pooled outcomes therefore characterize the overall clinical course after treatment but cannot establish efficacy specifically for erosion prevention or optical rehabilitation. The following sections therefore prioritize outcomes from EBMD eyes treated for recurrent erosions and identify mixed-etiology or mixed-indication evidence when stratified results are unavailable.

### 5.1. Epithelial Debridement and Manual Superficial Keratectomy

Simple epithelial debridement denotes removal of loose or poorly adherent epithelium without deliberate treatment of the exposed epithelial basement membrane interface. Manual superficial keratectomy additionally clears abnormal basement membrane and, when necessary, the superficial anterior stroma using a surgical blade. Procedural labels vary, so the operative steps matter more than the name [54].

A prospective study used IVCM to document epithelial and subbasal nerve changes after epithelial debridement followed by topical corticosteroid treatment [48]. The findings characterize structural changes during healing but do not establish EBMD-specific efficacy or the independent benefit of postoperative corticosteroids.

### 5.2. Diamond Burr Superficial Keratectomy (Polishing)

Diamond burr superficial keratectomy combines epithelial removal with controlled polishing of the exposed Bowman layer and, potentially, the superficial anterior stroma to eliminate residual abnormal interface material [54]. Unlike simple debridement, it intentionally modifies the epithelial adhesion substrate.

A randomized trial of 48 eyes with recurrent corneal erosions favored burr polishing over debridement alone, although traumatic and ABMD-associated cases were combined [55]. Long-term observational findings include a 91-eye series with 55 erosion-treated eyes, 36 associated with EBMD [42]. Recurrent erosion resolved in 97% of erosion-treated eyes with more than three months of follow-up (mean 33 months), a figure that pools EBMD-associated and post-traumatic cases [42].

Diamond burr polishing and PTK are supported options for central or diffuse EBMD. Neither an older ABMD-specific comparison nor a newer predominantly EBMD cohort establishes universal superiority of one procedure [43,45]. Diamond burr polishing does not require an excimer laser, whereas PTK permits controlled tissue removal and may also address optical irregularity. Counseling should address healing discomfort, recurrence, haze, and refractive consequences rather than characterize either procedure as curative [45,56].

### 5.3. Anterior Stromal Micropuncture: Focal Anchoring

Anterior stromal micropuncture, commonly termed ASP, was first described by McLean and colleagues [57]. It is commonly performed after debridement of loose or poorly adherent epithelium. Multiple small punctures are then placed through Bowman layer into the anterior stroma. The resulting focal fibrosis promotes epithelial adhesion rather than systematically removing the abnormal basement membrane across the affected cornea. Because puncture scars can impair visual quality, ASP is generally reserved for localized erosions outside the visual axis [52].

In a retrospective cohort of 35 eyes, 62.9% were free of recurrent erosion symptoms at a mean follow-up of 14 months; the remaining 37.1% had milder episodes, and 17% required additional intervention [58]. Visual fluctuation was not assessed as an outcome. Only one eye had known EBMD, whereas 62.9% had prior corneal trauma, so this cohort mainly reports post-traumatic RCES. A 2023 prospective study of 23 eyes included six with map-dot-fingerprint dystrophy [52]. Sixteen eyes required no further surgery, and IVCM documented keratocyte activation followed by scarring, consistent with the intended mechanism of focal scar-mediated adhesion.

The ASP literature derives largely from post-traumatic erosions, in which the adhesion defect is focal, whereas the interface abnormality in EBMD is often multifocal or diffuse. Nevertheless, EBMD itself is not a contraindication when the erosion is well localized and lies outside the visual axis. In the predominantly EBMD cohort reported by Yang and colleagues, recurrence was higher after debridement plus ASP than after burr polishing or PTK [43]. Because the study was nonrandomized and the reported rates were not EBMD-stratified, the findings do not establish universal inferiority of ASP for appropriately selected peripheral lesions. Peripheral ASP as an adjunct to central PTK is discussed below.

Nd:YAG laser stromal puncture has also been used to induce focal adhesion without direct needle penetration. A retrospective 33-eye series reported symptomatic improvement but included no ABMD cases [59]. This indirect evidence does not establish comparative effectiveness or general safety for central EBMD lesions.

### 5.4. Alcohol Delamination

Alcohol-assisted delamination uses dilute ethanol to loosen the corneal epithelium, which is then removed as a sheet without deliberate abrasion or ablation of Bowman layer. Electron microscopy demonstrates that the plane of separation varies with the underlying disorder. In 10 eyes with trauma-related recurrent erosions, separation occurred within the lamina lucida, leaving the lamina densa attached to the underlying cornea [15]. By contrast, in seven eyes with EBMD, the entire epithelial basal lamina was removed with the epithelial sheet. Thus, delamination may remove abnormal basement membrane as well as epithelium; a universal cleavage plane should not be assumed. Subsequent re-epithelialization is a repair response, not evidence that alcohol directly normalizes adhesion proteins. A small randomized comparison with PTK does not establish an EBMD-specific advantage [60].

### 5.5. PTK: Contemporary Evidence and Technical Caution

Conventional PTK follows epithelial removal and ablates the superficial corneal substrate, including any residual abnormal basement membrane, Bowman layer, and, depending on the protocol, anterior stroma. Treatment depth and optical zone vary; the intended effect is a more regular substrate for epithelial reattachment [38,49].

A 2023 meta-analysis reported a pooled recurrence rate of 18% after PTK, with substantial clinical heterogeneity [56]. Its corneal-dystrophy subgroup cannot be assumed to represent EBMD alone. Reported adverse outcomes included haze, hyperopic shift, and reduced corrected acuity. Disease-specific counseling is therefore better informed by cohorts that report painful erosions separately rather than combining multiple treatment indications.

Several studies provide more directly applicable evidence for EBMD-associated erosions. In the EBMD series reported by Lee and colleagues, 23 of 29 eyes treated for painful erosions initially responded and three responders subsequently recurred [38]. The estimated 88% five-year recurrence-free probability was conditional on initial treatment success and should not be interpreted as the success rate for all treated eyes [38]. Nevertheless, separation of the two indications makes this one of the more informative PTK series for EBMD-associated RCES. Yu and colleagues reported 40 patients with medically refractory EBMD treated with PTK over a follow-up window of 12–150 months [39]. One subsequent erosive event occurred and no patient underwent reoperation. This study extends disease-specific evidence, but its uncontrolled design and variable follow-up preclude treatment ranking or a universal ablation protocol.

Germundsson and colleagues evaluated 52 eyes with EBMD at a mean of 43 months after PTK (range, 7–100 months) [40]. No eye required retreatment, and five eyes (9.6%) experienced at least one mild or infrequent postoperative erosion. Symptomatic recurrence, defined as recurrent erosions or reduced vision, occurred in 7 eyes (13%), whereas morphologic recurrence on slit-lamp examination or IVCM occurred in 21 eyes (40%), indicating that morphologic recurrence does not necessarily correspond to clinical failure. Because only half of the eyes were treated for erosions and outcomes were not stratified by indication, these results characterize PTK in EBMD overall rather than EBMD-associated RCES specifically.

Transepithelial PTK uses the excimer laser to remove the epithelium as well as treat the underlying interface. Bizrah and colleagues reported 593 eyes, including 262 with ABMD [44]. The reported 75.8% rate of no ongoing treatment represented 91 of 120 telephone respondents rather than the full cohort. The findings suggest potential durability but do not provide an unbiased whole-cohort estimate of recurrence-free success.

Ablation depth may influence outcomes but remains incompletely defined. Gadhvi and colleagues reported better recurrence-free outcomes with at least 15 μm of stromal ablation than with shallower treatment [49]. Ablation depth was not randomized, and technique and case selection differed between groups. Moreover, total ablation depth during transepithelial PTK is not equivalent to stromal depth after mechanical epithelial removal. The experimental observation that deeper epithelial-stromal injury may delay EBM barrier recovery and favor TGF-β/PDGF-driven myofibroblast haze provides a countervailing concern, not an EBMD-specific depth rule [18,25]. These data do not support a universal threshold; depth should balance adequate interface removal against optical and stromal risks.

Treatment planning should distinguish erosion control from optical regularization. Prior corneal procedures, residual stromal thickness, refraction, treatment zone, ablation depth, and postoperative management may all affect healing, haze, and refractive change. These variables should be documented. A nominal ablation depth from one protocol should not be applied to another without accounting for these differences [38,39,,44,49].

### 5.6. Recurrence After a Procedure

Before repeat treatment, recurrent symptoms should be reassessed to distinguish renewed epithelial breakdown from residual optical irregularity or associated ocular surface disease. A retrospective salvage study compared a combined procedure (alcohol delamination, PTK, and peripheral ASP) with continued conservative management in eyes with RCE that had already failed prior surgery [61]. Recurrence occurred in 27.2% of the 92 operated eyes versus 69.6% in the 23 eyes in patients who declined reoperation, favoring the combined approach. Selection bias and the absence of established EBMD-specific effectiveness preclude designation of this combination as a preferred rescue procedure. The contribution of each component also cannot be isolated.

## 6. Optical Rehabilitation

Some patients with EBMD may not experience erosions and present only with blurred or fluctuating vision. This presentation is common in procedural series. Reduced vision was the sole indication for treatment in 26 of 52 eyes undergoing PTK in the series by Germundsson and colleagues [40]. In an earlier PTK series, 10 of 30 eyes were treated for erosions alone or in combination with visual symptoms, whereas the remaining 20 eyes were treated for visual symptoms alone [62]. In a debridement series of 74 eyes, visual difficulty was reported before treatment in 61 eyes (82%), whereas 13 eyes had erosion symptoms alone [46].

In patients treated for vision alone, the indication for treatment is visual rather than erosion-related, and treatment success is therefore assessed primarily by optical outcomes. Clinically significant central EBMD can alter cataract biometry even in the absence of recurrent pain. In one retrospective study, calculated spherical intraocular lens power changed after superficial keratectomy or PTK in 21 of 26 EBMD eyes [16]. The corneal surface should therefore be stabilized and keratometry and biometry repeated before final lens selection rather than relying on epithelial closure alone. A 2025 PTK cohort of 92 EBMD eyes documented improvements in visual acuity, higher-order aberrations, and corneal densitometry, but optical improvement and infrequent retreatment do not establish freedom from painful erosions, which remains a distinct outcome [41].

Before laser-assisted in situ keratomileusis, clinically significant EBMD warrants surface evaluation and treatment because epithelial sloughing during flap creation and epithelial ingrowth are concerns [63,64]; surface ablation may be preferable when refractive surgery is appropriate, and combined PTK/photorefractive keratectomy can address interface irregularity and refractive error in selected eyes. These decisions require individualized assessment rather than a universal procedure rule [3,18].

### Postoperative Amniotic Membrane

Amniotic membrane is a postoperative epithelial-healing adjunct after debridement or superficial keratectomy, not a method of optical regularization itself. Its basement membrane provides an adhesion substrate on which epithelial cells retain a progenitor phenotype through integrin-linked kinase-mediated signaling, which is the proposed mechanism for its support of epithelial recovery [65]. A small pre-cataract comparison found faster healing with cryopreserved membrane than with a bandage contact lens after debridement, without superior longer-term surface or visual stability [66]. In a 2026 retrospective uncontrolled series, 27 EBMD eyes underwent superficial keratectomy followed by cryopreserved amniotic membrane; epithelial healing and improved optical measures were documented over approximately two months [67]. Neither study establishes incremental durable erosion prevention. The membrane may be considered in selected eyes with impaired healing, but routine use requires comparative evidence.

## 7. Emerging Treatments and Mechanism-Guided Opportunities

Therapeutic potential can be considered along two dimensions: evidence of clinical benefit and evidence supporting the proposed mechanism. A clinically useful intervention may have an incompletely defined mechanism, whereas a biologically plausible target may lack a tested therapy. The following approaches therefore represent different stages of development and should not be interpreted as a uniform category of established EBMD treatments.

### 7.1. Topical Insulin

Topical insulin is a candidate therapy for supporting epithelial regeneration. A prospective nonrandomized comparison of 29 post-traumatic patients (14 conventional treatment vs. 15 with adjunctive insulin) reported improved healing and fewer recurrences with adjunctive insulin, but the report overlaps recurrent erosion and persistent epithelial defects in its terminology and outcomes [68]. The findings justify further investigation but do not establish efficacy in EBMD. Key questions include whether topical insulin improves epithelial attachment after closure and whether it provides incremental benefit after resurfacing. Controlled studies should use explicit disease phenotyping, standardized compounding and dosing, and recurrence follow-up rather than wound closure alone.

### 7.2. Neurotrophic and Matrix-Supporting Approaches

Cenegermin, a recombinant human nerve growth factor, improved epithelial healing in a randomized trial of neurotrophic keratopathy [69]. This establishes efficacy in neurotrophic keratopathy rather than EBMD-associated RCES. Neurotrophic support may represent a repurposing hypothesis for a defined subgroup with impaired epithelial recovery. Reduced corneal sensation should first prompt evaluation for coexisting neurotrophic disease. The trial does not support routine cenegermin use for otherwise typical EBMD erosions.

Heparan sulfate mimetic polymers (HSMPs) have both related-disease and direct EBMD evidence [70,71]. Labetoulle and colleagues, in a retrospective noninterventional series of 22 patients with EBMD presenting with spontaneous, recurrent acute ocular pain resistant to topical lubricants, added a topical HSMP while continuing lubricants [71]. Mean Ocular Surface Disease Index scores improved from 46.7 to 31.6 at the first visit and 32.5 at last follow-up (median 8.5 months). This uncontrolled symptomatic signal supports further evaluation of HSMPs as a comfort-directed adjunct but does not establish disease modification or durable prevention of erosions. A controlled EBMD trial should distinguish ocular discomfort from recurrent epithelial breakdown and assess incremental benefit beyond lubrication or resurfacing.

### 7.3. Differentiation, Adhesion, and Epithelial Repair Targets

Reduced PAX6 and desmoglein-1 expression in EBMD epithelium suggests differentiation and cell–cell cohesion as potential targets, but these associative findings also occur in other ocular surface disorders [23]. Desmoglein-1 mediates cell–cell adhesion rather than hemidesmosomal attachment. Topical fibronectin has been studied for persistent epithelial defects, including a double-masked randomized trial that found no benefit over vehicle, and paired substance P-derived FGLM-amide plus IGF-1-derived SSSR peptides have been studied in neurotrophic persistent defects [72,73]. Thymosin β4 and Rho-associated kinase inhibition are additional epithelial-healing research candidates, with evidence from other wound contexts or preclinical models [74,75]; none has established clinical efficacy for EBMD-associated recurrent erosion. Rho-associated kinase inhibition has context-dependent effects on epithelial migration, cell–matrix adhesion, and cell–cell junctions [76,77]. Improved wound closure in vitro does not establish restoration of durable hemidesmosomal attachment in EBMD. These interventions primarily target migration or wound closure, which must be distinguished from re-formation of stable EBM anchoring and long-term freedom from recurrence. Selective proteolysis control similarly requires disease- and phase-specific validation [26].

### 7.4. Rational Combination Treatment

Combination treatment has also been explored clinically. A retrospective sequential-cohort comparison reported recurrence in 1 of 25 eyes after central PTK plus peripheral ASP and in 5 of 23 eyes after PTK alone at approximately 20 months [78]. Only three eyes had ABMD, and use of historical treatment groups introduces confounding. The study nevertheless supports evaluation of an anatomically directed strategy that combines central regularization with peripheral anchoring. It does not establish routine prophylactic puncture for all eyes with EBMD. Transepithelial PTK combined with autologous serum was reported in 25 post-traumatic eyes, 20 of which had no further erosion during 6–20 months of follow-up [79]. Without a PTK-only comparator, the incremental contribution of serum remains uncertain. Resurfacing combined with serum, platelet-derived support, or selected anti-inflammatory treatment should therefore be evaluated as adjunctive strategies rather than presumed synergistic therapies. Comparative studies should standardize the underlying procedure and postoperative care while varying only the additional component. Table 2 summarizes adjunctive and emerging approaches, their primary targets, study designs and sample sizes, the certainty and applicability of the evidence, and priority clinical questions.

## 8. Research Priorities

Follow-up is most informative when it distinguishes initial epithelial closure from subsequent erosions, rescue medication or procedures, optical function, and complications. A clinically useful research outcome set could include time to first recurrence, episode severity, sleep disruption, patient-reported function, and clinician-confirmed epithelial defects over at least 12 months. This represents a proposed research framework rather than a validated universal follow-up schedule. Comparative studies require detailed reporting of the tissue-removal technique and relevant cointerventions rather than reliance on broad procedural labels such as debridement or superficial keratectomy. Studies should also report the etiology of erosions (EBMD, trauma, or both) and the indication for treatment (painful erosions, visual symptoms, or both), with outcomes stratified accordingly. Without this, EBMD-associated RCES cannot be separated from post-traumatic RCES or from EBMD treated for vision.

In clinical practice, early postoperative assessment focuses on confirmation of epithelial closure, the ongoing need for bandage contact lens protection, and detection of infection or delayed healing. Corticosteroid exposure also warrants intraocular pressure surveillance. Once the ocular surface has stabilized, persistent visual blur is best evaluated separately from recurrent pain, with repeat refraction as appropriate [3,38,41]. Patients require clear counseling that increasing pain, redness, or reduced vision during healing warrants prompt reassessment rather than presumptive attribution to another uncomplicated erosion.

## 9. Conclusions

RCES and EBMD are distinct entities, and the evidence that directly addresses their overlap is smaller than either literature alone suggests. Management of EBMD-associated RCES requires surface protection and durable epithelial attachment. Debridement removes unstable epithelium; manual superficial keratectomy, polishing, delamination, and PTK additionally address the interface in different ways. ASP instead induces focal adhesion-supporting fibrosis. Emerging therapies may complement these procedures, but their value requires patient-important outcomes and separation of demonstrated effects from indirect evidence and therapeutic hypotheses. The principal objective remains durable reduction in painful recurrences while preserving visual quality.

## Figures and Tables

**Figure 1:**
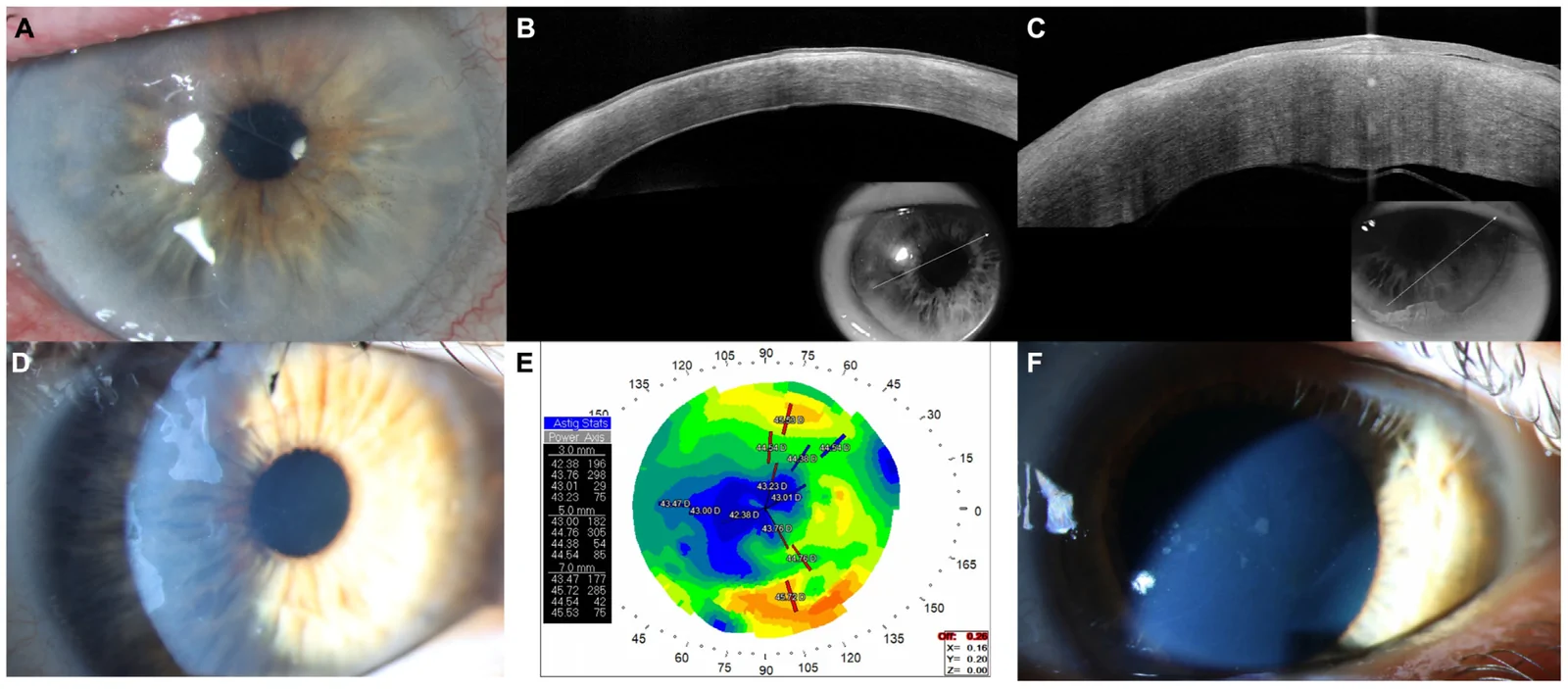
Clinical and multimodal imaging features of EBMD. (**A**) Slit-lamp photograph (BX 900, Haag-Streit AG, Köniz, Switzerland) showing fine, curvilinear, fingerprint-like epithelial lines. (**B**,**C**) Anterior segment optical coherence tomography (Avanti Scanner, Optovue, Fremont, CA, USA) scans demonstrating irregular, hyperreflective subepithelial changes with focal epithelial thickening and an uneven epithelial contour. Insets indicate the scan locations and orientations. (**D**) Slit-lamp photograph showing geographic, map-like epithelial opacities with irregular borders. (**E**) Axial corneal topography (TMS-4N, Tomey, Nagoya, Japan) demonstrating nonuniform corneal curvature, with asymmetric areas of flattening and steepening consistent with corneal surface irregularity. (**F**) Slit-lamp photograph showing scattered epithelial dots and faint curvilinear, map-like lines.

**Figure 2:**
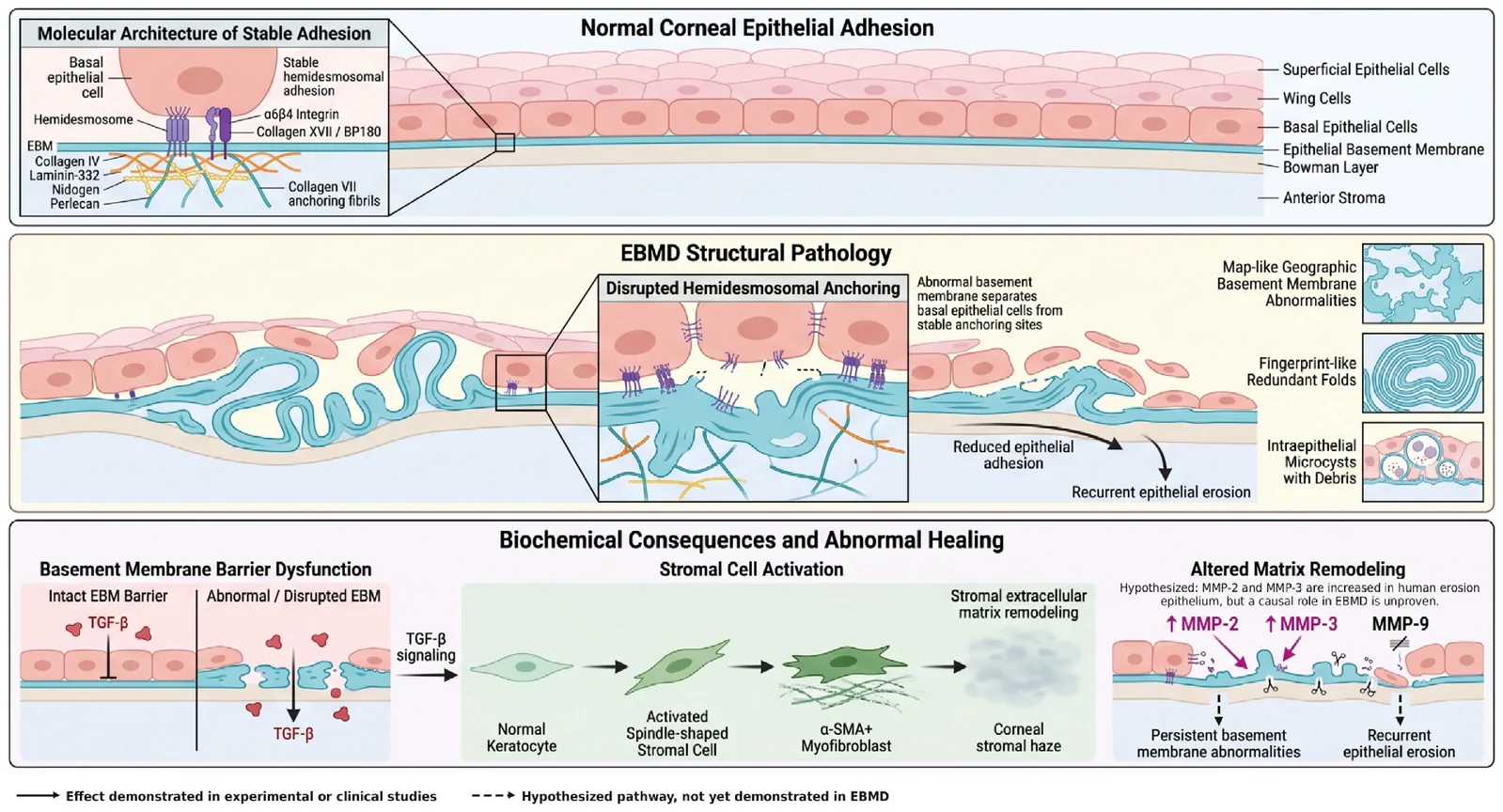
Biological mechanisms of epithelial basement membrane dystrophy: pathophysiology and cellular consequences. α-SMA, Alpha-smooth muscle actin; BP180, Bullous pemphigoid antigen 180-collagen XVII. (**Top**): normal adhesion complex. (**Middle**): multilaminar basement membrane extending into the epithelium with disrupted hemidesmosomal anchoring. (**Bottom**): loss of the EBM barrier permits TGF-β-driven myofibroblast transformation (demonstrated experimentally) and altered MMP activity is shown as a hypothesized contributor. Created in BioRender.

**Figure 3:**
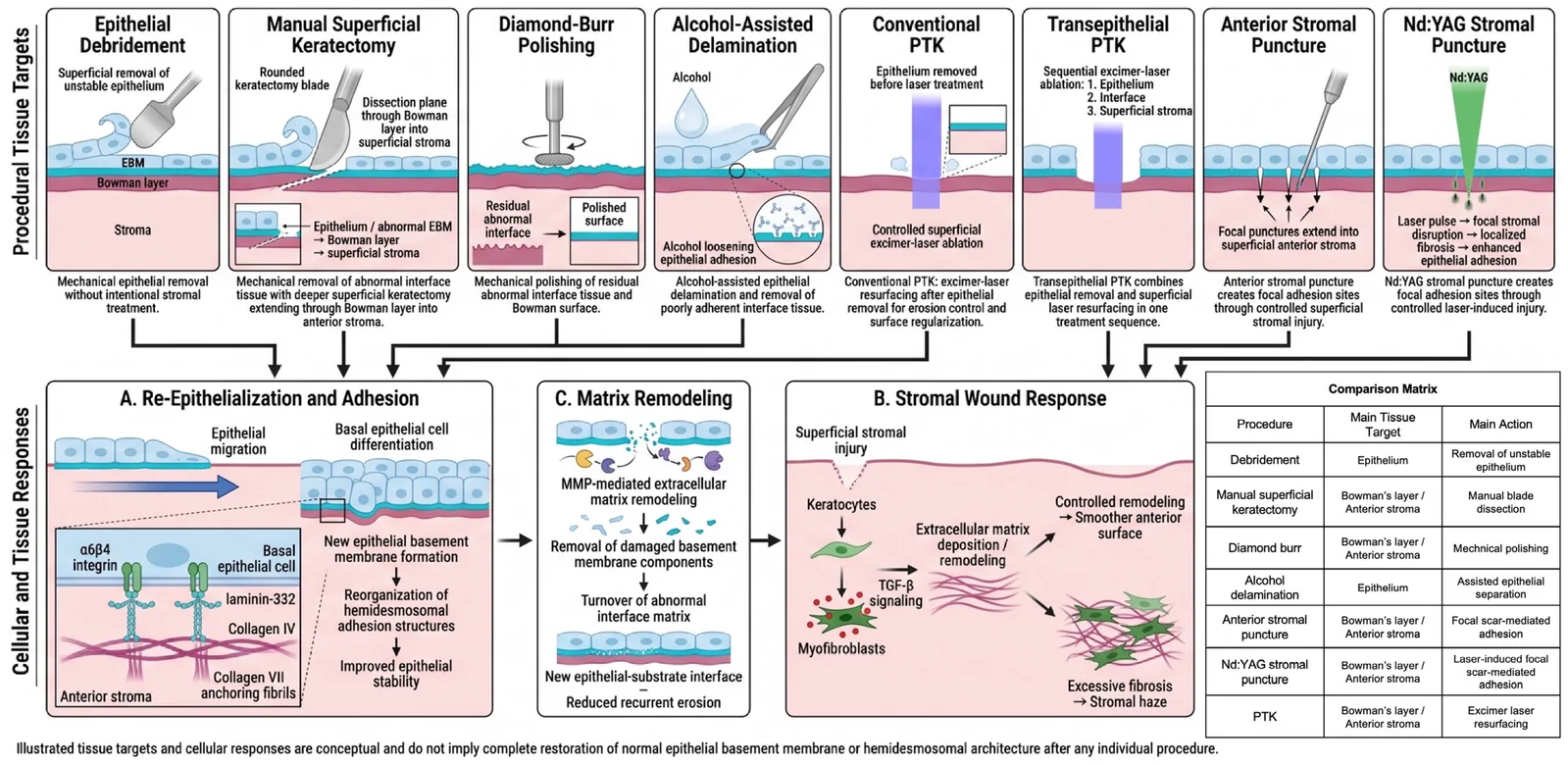
Procedural treatment of recurrent corneal erosions associated with epithelial basement membrane dystrophy. Upper row: tissue target of each procedure. Debridement and alcohol delamination leave Bowman layer intact; manual superficial keratectomy, diamond-burr polishing, and conventional and transepithelial PTK target Bowman layer and anterior stroma; anterior stromal and Nd:YAG puncture create focal breaches that heal by fibrosis. Lower panels: (**A**) re-epithelialization and adhesion; (**B**) stromal wound response; (**C**) MMP-mediated matrix remodeling (hypothesized). Responses are conceptual and do not imply complete restoration of normal EBM after any procedure. Created in BioRender. ASP, anterior stromal puncture; EBM, epithelial basement membrane; MMP, matrix metalloproteinase; PTK, phototherapeutic keratectomy.

**Table 1:** Clinical studies from the past 15 years (2011–2026) providing EBMD-specific evidence for treatment of recurrent corneal erosion, grouped by how directly their outcomes apply to EBMD-associated RCES.

Study	Design	Intervention vs. Comparator	Eyes, N (EBMD n); Indication	Follow-Up	Recurrence Definition	Recurrence, n/N (%) [95% CI] ^a^	Adverse Events
A. All-EBMD cohorts, erosion outcomes reported separately							
Lee 2017 [38]	Retrospective	PTK (mechanical epithelial removal); none	58 (58); erosion analysis 29, visual-symptom analysis 30	Erosion group mean 30.9 mo; overall cohort range 3–170 mo	Symptomatic recurrence of painful erosion ≥3 mo after successful PTK; morphologic recurrence	Initial nonresponse 6/29; recurrence after response 3/23, 13.0% (4.5–32.1); nonresponse or subsequent recurrence 9/29, 31.0% (17.3–49.2); morphologic 5/29; 5-y recurrence-free after response 88.0% (65.3–96.6), Kaplan–Meier	Haze 9/58 (15.5%); ≥2-line CDVA loss 1/29; no infections
Yu 2026 [39]	Retrospective, prospective outcome assessment	PTK, mean ablation 23 μm; none	40 (40); refractory painful erosions	12–150 mo	Erosive event; reoperation	1/40, 2.5% (0.4–12.9) (after minor trauma); reoperation 0/40	Grade ≥2 haze 0/40; refractive shift >0.5 D in 4/40; BCVA loss 1/40
B. All-EBMD cohorts, pooled indications (erosions and visual symptoms)							
Germundsson 2011 [40]	Cross-sectional follow-up with retrospective record review	PTK; none	52 (52); erosions 18, visual 26, both 8	Mean 43 mo (7–100)	Symptomatic (erosions or reduced vision); morphologic (slit lamp or IVCM)	Symptomatic 7/52, 13.5% (6.7–25.3); ≥1 erosive episode 5/52, 9.6% (4.2–20.6); morphologic 21/52, 40.4% (28.2–53.9); reoperation 0/52 included eyes ^b^	Mean hyperopic shift 0.49 D; BSCVA unchanged or improved 49/51
Helm 2025 [41]	Retrospective	PTK, mean ablation 14 μm, mitomycin C; none	92 (92); irregular astigmatism, reduced visual quality, or erosions	Mean 331.6 d (4–2867 d)	Reported recurrence; re-PTK for residual scars/irregular astigmatism; slit-lamp residuum	One reported recurrence/re-PTK: 1/92, 1.1% (0.2–5.9), using total cohort; residuum reported in 19 eyes (21.1%), 6 eyes (31.6%) beyond 1 y ^c^	Mild haze in 9 eyes (reported 10.0%); no moderate/severe haze; dry eye/punctate keratitis in 17 eyes (reported 18.9%) ^c^
C. Mixed cohorts with an EBMD denominator							
Vo 2015 [42]	Retrospective	Epithelial debridement + diamond burr; none	91 (36 of 55 erosion-treated); erosions 55, visual 42, both 6	Erosion group mean 25.2 mo (≥1 mo, n = 49); 33.2 mo (≥3 mo, n = 37)	Recurrent erosion	2/49, 4.1% (1.1–13.7), with ≥1 mo follow-up; both recurrences in EBMD eyes without trauma ^d^	Persistent haze at final follow-up: 7/49 (14.3%) in erosion group; 1 staphylococcal marginal keratitis; 1 HSV keratitis; ≥2-line CDVA loss 3/80 (2 unrelated; 1 progression of pre-existing edema)
Tsatsos 2022 [32]	Prospective, uncontrolled	5% NaCl ointment twice daily for 30 d after acute episode; none	21 (12); acute erosion	6 mo	New erosion episode	ABMD 0/12, 0.0% (0.0–24.2); post-traumatic 1/9 (after new trauma)	No scarring or keratitis; endothelial cell counts stable
Yang 2023 [43]	Retrospective, nonrandomized	Debridement + ASP (34) vs. debridement + diamond burr (33) vs. PTK (30)	97 (78); erosions; EBMD by arm: ASP 22/34, burr 26/33, PTK 30/30	Mean 474 d; median ASP 189 d, burr 64 d, PTK 542 d	Objective epithelial defect or symptoms of erosion	ASP 10/34, 29.4% (16.8–46.2); burr 3/33, 9.1% (3.1–23.6); PTK 3/30, 10.0% (3.5–25.6; all EBMD). Overall, 14/16 recurrences in EBMD; EBMD recurrence counts not separated for ASP/burr	Mild haze 2 (1 burr, 1 PTK); hyperopic shift 1 D in 2 PTK eyes; no infiltrates
Bizrah 2024 [44]	Retrospective chart review + telephone survey	Transepithelial PTK, 9 mm total zone, mitomycin C; none ^e^	593 (262); RCES refractory to ≥3 conservative treatments	Mean 60.5 mo (5–127)	Repeat PTK for persistent/recurrent symptoms	Repeat PTK overall 6/593, 1.0% (0.5–2.2); EBMD subgroup 3/262, 1.1% (0.4–3.3), derived from reported counts	Grade 2 haze 9/593 (1.5%), none grade ≥3; CDVA loss reported in 20% of 126-eye acuity sample (mean loss 1.5 lines)

^a^ Percentages and Wilson 95% confidence intervals were calculated from reported counts, except Lee’s 5-year recurrence-free estimate and confidence interval, which were reported using Kaplan–Meier analysis. Outcomes, denominators, follow-up periods, and haze thresholds differ across studies and should not be directly compared. Group A reports erosion outcomes in all-EBMD cohorts; group B pools indications; group C includes mixed cohorts, with EBMD-specific erosion results available for Tsatsos and Yang’s PTK arm and an EBMD-specific retreatment estimate derivable for Bizrah. ^b^ Germundsson excluded one additional eye reoperated for persistent subepithelial haze. ^c^ Helm’s complication percentages imply 90 evaluable eyes despite a 92-eye cohort; denominators are unclear. Residual or recurrent EBMD is not an erosion endpoint. ^d^ Vo also reported 2/37 in a longer-follow-up analysis, but its footnote included one early recurrence with <3 months’ follow-up; the ≥1-month estimate is presented here. ^e^ Bizrah’s Methods specify 50 μm epithelial removal plus 15 μm subepithelial treatment; the Discussion states 10 μm stromal ablation. Earlier EBMD series ([16,45,46]) and studies without an EBMD denominator are discussed in the text. ABMD, anterior basement membrane dystrophy; ASP, anterior stromal puncture; BCVA/BSCVA/CDVA, best (spectacle-)corrected (distance) visual acuity; CI, confidence interval; EBMD, epithelial basement membrane dystrophy; HSV, herpes simplex virus; IVCM, in vivo confocal microscopy; PTK, phototherapeutic keratectomy.

**Table 2:** Adjunctive and emerging approaches: proposed actions, study designs and sample sizes, evidence applicability, and clinical research questions.

Approach	Proposed Action (Primary Target)	Study Design and Sample Size	Applicability to EBMD-Associated RCES	EBMD-Centered Research Question
Oral doxycycline + topical corticosteroid	Inflammatory and proteolytic modulation (inflammation/proteolysis)	Retrospective case series of recalcitrant recurrent erosions, 7 patients; no comparator [35].	None; no EBMD patients	Does treatment reduce recurrence in inflammatory EBMD phenotypes?
Autologous serum	Epithelial repair support (epithelial closure)	Prospective uncontrolled series, 33 eyes (serum alone); uncontrolled series, 25 post-traumatic eyes (serum after transepithelial PTK) [37,79].	None; EBMD outcomes not reported separately	Does serum add benefit after standardized resurfacing?
Platelet-rich plasma	Epithelial repair support (epithelial closure)	Retrospective historical-control comparison, 47 eyes (27 platelet-rich plasma vs. 20 lubrication only) [36].	Minimal; 3 EBMD eyes (1 treated, 2 control) in a mixed cohort	Does benefit extend to a defined EBMD population?
Amniotic membrane	Postoperative surface protection (epithelial closure)	Pilot comparison, 19 eyes (9 prospective cryopreserved membrane vs. 10 retrospective bandage lens); single-arm series, 27 eyes, approximately 2-month follow-up [66,67].	Direct; EBMD eyes; no recurrence outcomes, short follow-up	Which patients benefit beyond standard postoperative care?
Heparan sulfate mimetic polymer	Matrix support (adhesion substrate; mechanism unverified in EBMD)	Retrospective noninterventional series, 22 patients; median follow-up 8.5 months [71].	Direct; EBMD patients; symptom scores only, no erosion counts	Does benefit include fewer confirmed erosions?
Topical insulin	Epithelial regeneration (epithelial closure)	Prospective nonrandomized two-group comparison; 29 post-traumatic patients (14 conventional treatment vs. 15 with adjunctive insulin); single center [68].	None; no EBMD patients	Does improved healing translate into durable epithelial stability?
Cenegermin	Neurotrophic support (epithelial closure)	Multicenter randomized vehicle-controlled trial, 48 patients with neurotrophic keratopathy (24 per arm) [69].	Indirect; neurotrophic keratopathy; no EBMD patients	Could selected EBMD patients with impaired healing benefit?
PAX6 and desmoglein-1 targets	Differentiation and cell–cell adhesion (adhesion substrate; preclinical)	Laboratory expression study (qPCR, Western blot) of corneal epithelium, 5 EBMD patients plus disease and healthy controls [23].	Direct (tissue); EBMD specimens; associative, no therapy tested	Would pathway restoration improve epithelial stability?
Topical fibronectin	Adhesion substrate for epithelial migration (epithelial closure)	Double-masked randomized trial, 65 patients with persistent epithelial defects (20 fibronectin, 23 vehicle, 22 placebo); no benefit over vehicle [72].	Indirect; persistent epithelial defects; negative result	Does substrate support after resurfacing improve epithelial anchoring in EBMD?
Substance P- and insulin-like growth factor-1-derived peptides (FGLM-amide + SSSR)	Neurotrophic support of epithelial migration (epithelial closure)	Open-label uncontrolled study, 26 eyes with neurotrophic persistent epithelial defects; closure in 19 (73%) within 4 weeks [73].	Indirect; neurotrophic defects; no EBMD patients	Could EBMD patients with reduced corneal sensation benefit?
Thymosin β4	Epithelial migration and wound closure (epithelial closure)	Uncontrolled case series, 9 patients with chronic neurotrophic epithelial defects [74].	Indirect; neurotrophic defects; no EBMD patients	Does faster closure translate into stable basement membrane anchoring?
Rho-associated kinase inhibitors	Cytoskeletal modulation of epithelial migration (epithelial closure)	Two in vitro human corneal epithelial cell studies (no patients) [76,77]; one neurotrophic-ulcer case report (1 patient) [80]	None; in vitro and single case report; no EBMD patients	Would Rho-kinase inhibition improve epithelial attachment rather than closure alone?

Certainty of evidence is indicated by the study design column (in vitro study, case report, uncontrolled series, nonrandomized comparison, or randomized trial). Applicability indicates how directly the evidence bears on EBMD-associated RCES: direct (EBMD patients or tissue), minimal (few EBMD eyes within a mixed cohort), indirect (a different disease), or none (no EBMD patients). Questions are research proposals, not treatment recommendations. EBMD, epithelial basement membrane dystrophy; PTK, phototherapeutic keratectomy; qPCR, quantitative polymerase chain reaction.

## Data Availability

No new data were created or analyzed in this study. Data sharing is not applicable to this article.

## Abbreviations

The following abbreviations are used in this manuscript:

ABMD	Anterior basement membrane dystrophy
ASP	Anterior stromal puncture
EBM	Epithelial basement membrane
EBMD	Epithelial basement membrane dystrophy
HSMP	Heparan sulfate mimetic polymer
IGF-1	Insulin-like growth factor 1
IVCM	In vivo confocal microscopy
MMP	Matrix metalloproteinase
Nd:YAG	Neodymium-doped yttrium aluminum garnet
PAX6	Paired box 6
PDGF	Platelet-derived growth factor
PTK	Phototherapeutic keratectomy
qPCR	Quantitative polymerase chain reaction
RCE	Recurrent corneal erosion
RCES	Recurrent corneal erosion syndrome
TGF-β	Transforming growth factor beta
AS-OCT	Anterior segment optical coherence tomography
